# Projection-Based DMRG-in-DFT Embedding Corrected by Nonadditive Exchange-Correlation

**DOI:** 10.1021/acs.jctc.5c01930

**Published:** 2026-02-09

**Authors:** Enzo Monino, Daria Drwal, Pavel Beran, Michał Hapka, Libor Veis, Katarzyna Pernal

**Affiliations:** † 86875 J. Heyrovský Institute of Physical Chemistry, Academy of Sciences of the Czech Republic, v.v.i., Dolejškova 3, 18223 Prague 8, Czech Republic; ‡ Institute of Physics, Lodz University of Technology, ul. Wolczanska 217/221, 93-005 Lodz, Poland; § Faculty of Mathematics and Physics, Charles University, 12116 Prague, Czech Republic; ∥ 49605 University of Warsaw, Faculty of Chemistry, ul. L. Pasteura 1, 02-093 Warsaw, Poland

## Abstract

The projection-based wave function in density functional theory (WF-in-DFT) embedding enables an efficient description of both the energetics and properties of large and complex chemical systems, with accuracy exceeding that of pure DFT. Recently, we have proposed using the density matrix renormalization group (DMRG) as the WF method for molecules containing strongly correlated fragments [ BeranP.,  J. Phys. Chem. Lett. 2023, 14, 716–722 36648273 10.1021/acs.jpclett.2c03298PMC10017021]. In this work, we demonstrate that the accuracy of the DMRG-in-DFT approach is primarily limited by the approximate treatment of the coupling between the active component and its environment through nonadditive exchange-correlation functionals. To address this issue, we combine exact exchange to reduce the nonadditive exchange error with a multireference adiabatic connection (AC) scheme to recover nonadditive correlation. The performance of the improved DMRG-in-DFT embedding is illustrated on two prototypical strongly correlated systems: the dissociation of the H_20_ chain and the cleavage of a triple CN bond in propionitrile.

## Graphical abstract

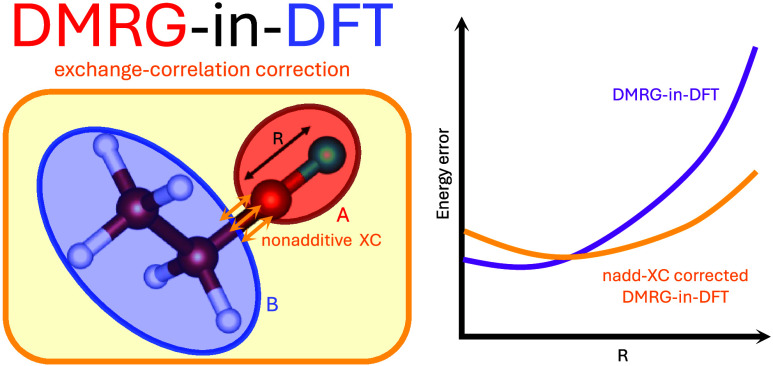

## 1 Introduction

Strong electron correlation underpins many essential phenomena in chemistry, influencing bond dissociation, open-shell and excited-state electronic structures, and catalytic reactivity.^1,2^ Despite its fundamental importance, accurately and efficiently describing strongly correlated systems remains a major challenge in quantum chemistry. In principle, full configuration interaction (FCI) provides an exact solution to the electronic Schrödinger equation within a given one-particle basis, but its exponential scaling makes it impractical for all but the smallest systems. Consequently, a variety of approximate, polynomially scaling wave function (WF) methods have been developed, each systematically improvable toward the FCI limit. For systems dominated by weak correlation, the coupled-cluster (CC) framework^3^ remains the method of choice. In contrast, molecules exhibiting strong (static) correlation, such as transition-metal complexes or those undergoing bond dissociation, are more appropriately treated within the complete active space (CAS) framework,^4^ which forms the conceptual basis for this work.

The complete active space self-consistent field (CASSCF) method^5^ combines a FCI treatment within a chosen active orbital space with orbital optimization, providing the standard starting point for multireference (MR) calculations. To recover the missing dynamical correlation, post-SCF approaches such as complete active space second-order perturbation theory (CASPT2),^6^ *n*-electron valence state perturbation theory (NEVPT2),^7^ multireference adiabatic connection (AC)^8,9^ or multireference configuration interaction (MRCI)^2^ are employed. However, all of these methods are fundamentally limited by the exponential scaling of the FCI problem within the active space, restricting practical CAS sizes to maximally 20 orbitals.^10^

To address this limitation, several approximate FCI solvers have been introduced. Among them, the density matrix renormalization group (DMRG) method^11^ has become one of the most powerful techniques for treating strong correlation. Since its introduction into quantum chemistry,^12^ DMRG has proven capable of accurately describing systems with dozens of active orbitals.^13–16^ This success has led to the development of a number of post-DMRG approaches designed to recover out-of-CAS dynamical correlation.^17^ Despite the availability of methods that allow for an efficient treatment of dynamic correlation in large active spaces,^18^ wave function-based approaches remain computationally demanding for large systems.

In contrast, density functional theory (DFT) provides a computationally efficient framework applicable to very large molecules. In its most common framework, namely Kohn–Sham (KS) DFT, the accuracy of DFT is limited by generic deficiency of most workable approximations to exchange-correlation (xc) functionals. Basically, their single-reference character limits reliability of KS-DFT for strongly correlated problems.^19^

A promising route toward combining the accuracy of WF-based methods with the efficiency of DFT lies in quantum embedding frameworks.^20^ These approaches exploit the locality of electronic interactions by partitioning a molecular system into an active region treated with a high-level method and an environment treated with a lower-level one.^20,21^ Neugebauer, Reiher, and co-workers introduced the first DMRG-in-DFT embedding scheme based on the frozen density embedding (FDE) formalism.^22^ However, due to the approximate nature of the nonadditive kinetic potential (NAKP), their proof-of-concept studies were limited to systems in which the active region was not covalently bonded to its environment.

The projection-based DFT (PB-DFT) embedding method^23,24^ overcomes the limitations of the FDE framework by enforcing orthogonality between the occupied orbitals of the subsystems through a level-shift projection operator, thereby removing the need for an approximate NAKP. Related parameter-free formulations have also been proposed.^25,26^ The PB-DFT approach is formally exact in the sense that when both subsystems are described at the same DFT level, their combined energy reproduces the total energy of the full system. Its true power, however, emerges in WF-in-DFT embedding schemes, where a high-level wave function method is applied to the active region while the surrounding environment is treated efficiently with DFT.

Building on the demonstrated success of PB-DFT embedding across diverse applications, including transition-metal catalysis, enzymatic reactivity, and electrolyte decomposition,^27–30^ and leveraging the proven capability of DMRG to capture strong static correlation, we have previously developed the projection-based DMRG-in-DFT embedding framework.^31^ This method can offer an accurate and computationally efficient approach for describing extended molecular systems with strongly correlated fragments within a multiscale electronic structure framework. Alternative multireference PB-DFT embedding approaches comprise CASSCF-in-DFT,^32^ or CASPT2-in-DFT.^26^

We note that complementary embedding strategies include, for example, self-energy embedding theory (SEET)^33^ and the subsystem embedding subalgebra formalism,^34^ which underpin recent developments in active-space coupled-cluster downfolding techniques.^35^

Goodpaster et al.^24^ analyzed the sources of error in projection-based (PB) WF-in-DFT embedding and identified the dominant contribution as the nonadditive XC error arising from the approximate nature of DFT functionals. To mitigate this problem, they proposed replacing the DFT-based nonadditive XC energy with one evaluated at the second-order Møller−Plesset (MP2) level. In related work, Bensberg and Neugebauer^36^ demonstrated, using the Cr­(CO)_6_ complex as a representative transition-metal system, that the apparent accuracy of DFT-in-DFT embedding often stems from a delicate balance of error cancellation. They emphasized that simply substituting the DFT description of the active subsystem with a high-level WF treatment does not necessarily improve reaction energies or barriers unless the interaction energy between subsystems is also refined. This issue is expected to become even more pronounced for systems with strongly correlated active regions, where nonadditive XC errors can be substantial. In this work, we address this challenge and improve the DMRG-in-DFT method by introducing corrections to the nonadditive exchange and correlation energies, with the latter derived from the genuinely multireference adiabatic connection (AC) framework.^8,9^

The paper is organized as follows. In Section 2, we briefly summarize the DMRG-in-DFT embedding framework and outline the computation of nonadditive exchange−correlation corrections using the multireference AC approach.^8,9^ Section 3 presents the computational details of the benchmark calculations, and the corresponding results are discussed in Section 4. Finally, conclusions are given in Section 5.

## 2 Theory

In the projection-based DMRG-in-DFT^31^ the total energy of a composite system AB reading

1 $$E_{\mathrm{DMRG}‐\mathrm{in}‐\mathrm{DFT}}=E_{\mathrm{DMRG}}\left[\Psi^{A}\right]+E_{\mathrm{DFT}}\left[\gamma^{A}+\gamma^{B}\right]-E_{\mathrm{DFT}}\left[\gamma^{A}\right]+\mathrm{tr}\left[\left(\gamma_{\mathrm{emb}}^{A}-\gamma^{A}\right)\upsilon_{\mathrm{emb}}\left[\gamma^{A},\gamma^{B}\right]\right]$$

is given in terms of density matrices of the subsystems, γ^A^ and γ^B^, following from KS-DFT computation carried out in the first step of the embedding procedure. These density matrices are defined by *N* orthogonal spinorbitals {φ_*i*_}_*i*=1...*N*_, *N* indicating the number of electrons in the composite system, assigned to one of the subsystems comprising *N*_*X*_ (X = A, B) electrons

2 $$\gamma^{A}\left(x,x^{\prime }\right)=\sum_{i\in A}^{N_{A}}\varphi_{i}\left(x\right)\varphi_{i}\left(x^{\prime }\right)$$

3 $$\gamma^{B}\left(x,x^{\prime }\right)=\sum_{i\in B}^{N_{B}}\varphi_{i}\left(x\right)\varphi_{i}\left(x^{\prime }\right)$$

In addition to γ^A^ and γ^B^, the computation of the DMRG-in-DFT energy according to eq 1 requires a third ingredient, namely the wave function Ψ^A^. The corresponding *E*_DMRG_[Ψ^A^] energy is computed as

4 $$E_{\mathrm{DMRG}}\left[\Psi^{A}\right]=\left\langle \Psi^{A}|\sum_{\sigma }\sum_{\mathrm{pq}}h_{\mathrm{pq}}{â}_{p_{\sigma }}^{†}{â}_{q_{\sigma }}+\frac{1}{2}\sum_{\sigma \sigma \prime }\sum_{\mathrm{pqrs}}\left\langle \mathrm{pq}|\mathrm{rs}\right\rangle {â}_{p_{\sigma }}^{†}{â}_{q_{\sigma \prime }}^{†}{â}_{s_{\sigma \prime }}{â}_{r_{\sigma }}|\Psi^{A}\right\rangle$$

where *p*, *q*, *r*, *s* are indices of general orbitals, *p*_σ_ denotes a spinorbital. The one-electron Hamiltonian operator

5 $$ĥ=-\frac{1}{2}\Delta -\sum_{I}\frac{Z_{I}}{\left|r-R_{I}\right|}$$

contains the kinetic energy and electron−nucleus attraction terms, where the summation extends over all nuclei *I* in the composite system.

The wave function Ψ^A^ results from a DMRG calculation on the subsystem A. It is carried out with the Hamiltonian *Ĥ* ^A^, which includes electron−electron interactions within the subsystem A and interaction energy of electrons with all nuclei in the composite system, and an embedding potential υ̂^emb^, accounting for other than electron-nuclei interactions of the subsystems. In the language of second quantization, *Ĥ* ^A^ acts in the space spanned by states constructed from spinorbitals belonging to the subset assigned to A

6 $${Ĥ}^{A}=\sum_{\sigma }\sum_{\mathrm{pq}\in A}\left(h_{\mathrm{pq}}+\upsilon_{\mathrm{pq}}^{\mathrm{emb}}\right){â}_{p_{\sigma }}^{†}{â}_{q_{\sigma }}+\frac{1}{2}\sum_{\sigma \sigma \prime }\sum_{\mathrm{pqrs}\in A}\left\langle \mathrm{pq}|\mathrm{rs}\right\rangle {â}_{p_{\sigma }}^{†}{â}_{q_{\sigma \prime }}^{†}{â}_{s_{\sigma \prime }}{â}_{r_{\sigma }}$$

The local embedding potential υ̂^emb^ is a bifunctional depending on density matrices of both subsystems and it is defined as a sum of Hartree (*H*), exchange (*x*), and correlation (*c*) interaction potentials. It can be written as

7 $${\mathrm{\upsilon ̂}}^{\mathrm{emb}}\left[\gamma^{A},\gamma^{B}\right]\left(r\right)=\int \frac{\rho^{B}\left(r^{\prime }\right)}{\left|r-r^{\prime }\right|}dr^{\prime }+{\mathrm{\upsilon ̂}}_{\mathrm{xc}}\left[\gamma^{A}+\gamma^{B}\right]\left(r\right)-{\mathrm{\upsilon ̂}}_{\mathrm{xc}}\left[\gamma^{A}\right]\left(r\right)$$

where the first term represents the Hartree potential generated by the electron density of subsystem B, ρ^B^(**r**). Notice that in a representation of general orbitals {φ_*p*_(**r**)}, electron density is determined by a density matrix γ

8 $$\rho \left(r\right)=\sum_{\mathrm{pq}}\sum_{\sigma }\gamma_{p_{\sigma }q_{\sigma }}\varphi_{p}\left(r\right)\varphi_{q}\left(r\right)$$

The operator *v̂*_*xc*_ denotes the sum of exchange and correlation potentials, *v̂*_*xc*_ = *v̂*_*x*_ + *v̂*_*c*_. These potentials are typically derived from the exchange-correlation functional *E*_*xc*_[ρ] employed in the initial KS-DFT calculation of the embedding procedure, according to

9 $${\mathrm{v̂}}_{\mathrm{xc}}\left[\rho \right]=\frac{\delta E_{\mathrm{xc}}\left[\rho \right]}{\delta \rho }$$

The last term of the *E*_DMRG−in−DFT_ expression, eq 1, involving the density-matrix difference γ_emb_^A^ − γ^A^, accounts for the lack of self-consistency between the density matrix used to construct the embedding potential and the one resulting from the DMRG computation. A fully self-consistent procedure, denoted here as SCF-DMRG-in-DFT, in which γ^A^ is iteratively updated in υ̂^emb^, would allow this correction to be omitted. For the sake of compactness, this term will be dropped in all subsequent equations, regardless of whether we refer to DMRG-in-DFT or SCF-DMRG-in-DFT.

In order to recast the total energy expression, eq 1, into an equivalent form that explicitly separates the nonadditive contributions we aim to correct, let us first recall that within the KS-DFT framework the energy functional is given by

10 $$E^{\mathrm{DFT}}\left[\rho \right]=T_{s}\left[\rho \right]+E_{\mathrm{ext}}\left[\rho \right]+E_{H}\left[\rho \right]+E_{\mathrm{xc}}\left[\rho \right]$$

where *T_s_* [ρ] denotes the noninteracting kinetic-energy functional, *E*_ext_[ρ] the interaction with the external potential, *E_H_* [ρ] the Hartree energy, and *E_xc_* [ρ] the exchange-correlation functional. In KS-DFT, the noninteracting reference system is described by a set of orthogonal spinorbitals {φ_*i*_} that reproduce the electron density ρ. The corresponding noninteracting kinetic-energy functional is defined explicitly in terms of the KS spinorbitals as

11 $$T_{s}\left[\rho \right]=T_{s}\left[\gamma \right]=-\frac{1}{2}\sum_{i}^{N}\left\langle \varphi_{i}\right|\Delta \left|\varphi_{i}\right\rangle$$

where the one-particle density matrix is given by γ­(*x*, *x*′) = ∑_*i*_^*N*^φ_*i*_(*x*)­φ_*i*_(*x*′). Using the orthogonality of the orbitals assigned to subsystems A and B, which define the density matrices γ^A^ and γ^B^ (recall that these orbitals originate from a single KS-DFT calculation of the entire system and are therefore orthogonal), together with eq 11 and the linearity of the functional *E*_ext_[ρ], we obtain

12 $$E_{\mathrm{ext}}\left[\rho^{A}+\rho^{B}\right]=\int \left(\rho^{A}\left(r\right)+\rho^{B}\left(r\right)\right)\upsilon_{\mathrm{ext}}\left(r\right)dr=E_{\mathrm{ext}}\left[\rho^{A}\right]+E_{\mathrm{ext}}\left[\rho^{B}\right]$$

so that the energy *E*_DFT_[γ^A^ + γ^B^] appearing in eq 1 can be decomposed as follows

13 $$E_{\mathrm{DFT}}\left[\gamma^{A}+\gamma^{B}\right]=T_{s}\left[\gamma^{A}\right]+T_{s}\left[\gamma^{B}\right]+E_{\mathrm{ext}}\left[\gamma^{A}\right]+E_{\mathrm{ext}}\left[\gamma^{B}\right]+E_{H}\left[\gamma^{A}+\gamma^{B}\right]+E_{\mathrm{xc}}\left[\gamma^{A}+\gamma^{B}\right]$$

Consequently, the difference between the DFT energy of the composite system and that of subsystem A is expressed as the sum of the environment energy B and the Hartree and exchange-correlation nonadditive terms. Namely

14 $$E_{\mathrm{DFT}}\left[\gamma^{A}+\gamma^{B}\right]-E_{\mathrm{DFT}}\left[\gamma^{A}\right]=E_{\mathrm{DFT}}\left[\gamma^{B}\right]+E_{H}^{\mathrm{nadd}}\left[\gamma^{A},\gamma^{B}\right]+E_{\mathrm{xc}}^{\mathrm{nadd}}\left[\gamma^{A},\gamma^{B}\right]$$

where the nonadditive Hartree energy functional reads

15 $$E_{H}^{\mathrm{nadd}}\left[\gamma^{A},\gamma^{B}\right]\equiv E_{H}\left[\gamma^{A}+\gamma^{B}\right]-E_{H}\left[\gamma^{A}\right]-E_{H}\left[\gamma^{B}\right]=\int \int \frac{\rho^{A}\left(r\right)\rho^{B}\left(r^{\prime }\right)}{\left|r-r^{\prime }\right|}drdr^{\prime }$$

*E*_*xc*_^nadd^ is defined as the sum of the nonadditive exchange and correlation functionals

16 $$E_{\mathrm{xc}}^{\mathrm{nadd}}\left[\gamma^{A},\gamma^{B}\right]=E_{x}^{\mathrm{DFT},\mathrm{nadd}}\left[\gamma^{A},\gamma^{B}\right]+E_{c}^{\mathrm{DFT},\mathrm{nadd}}\left[\gamma^{A},\gamma^{B}\right]$$

where

17 $$E_{x}^{\mathrm{DFT},\mathrm{nadd}}\left[\gamma^{A},\gamma^{B}\right]\equiv E_{x}\left[\gamma^{A}+\gamma^{B}\right]-E_{x}\left[\gamma^{A}\right]-E_{x}\left[\gamma^{B}\right]$$

and analogously for the correlation functional *E*_*c*_^DFT,nadd^. Both the nonadditive exchange and correlation functionals are approximate and thus inherit the errors of the underlying exchange and correlation functionals.

With the above definitions of the nonadditive functionals, the total energy, eq 1, can be rewritten in the equivalent form

18 $$E_{\mathrm{DMRG}‐\mathrm{in}‐\mathrm{DFT}}=E_{\mathrm{DMRG}}\left[\Psi^{A}\right]+E_{\mathrm{DFT}}\left[\gamma^{B}\right]+E_{H}^{\mathrm{nadd}}\left[\gamma^{A},\gamma^{B}\right]+E_{c}^{\mathrm{DFT},\mathrm{nadd}}\left[\gamma^{A},\gamma^{B}\right]+E_{x}^{\mathrm{DFT},\mathrm{nadd}}\left[\gamma^{A},\gamma^{B}\right]$$

If Ψ^A^ is obtained from a self-consistent procedure, it may be more appropriate to evaluate the nonadditive energy terms with γ_emb_^A^, which leads to

19 $$E_{\mathrm{SCF}‐\mathrm{DMRG}‐\mathrm{in}‐\mathrm{DFT}}=E_{\mathrm{DMRG}}\left[\Psi^{A}\right]+E_{\mathrm{DFT}}\left[\gamma^{B}\right]+E_{H}^{\mathrm{nadd}}\left[\gamma_{\mathrm{emb}}^{A},\gamma^{B}\right]+E_{c}^{\mathrm{DFT},\mathrm{nadd}}\left[\gamma_{\mathrm{emb}}^{A},\gamma^{B}\right]+E_{x}^{\mathrm{DFT},\mathrm{nadd}}\left[\gamma_{\mathrm{emb}}^{A},\gamma^{B}\right]$$

The nonadditive exchange and correlation energy functionals describe the exchange and correlation between subsystems A and B. When semilocal functionals are applied to strongly correlated systems, they introduce significant static correlation errors. Our goal is to correct the nonadditive exchange-correlation contributions.

Exploiting the orthogonality of the orbitals used to construct γ_emb_^A^ and γ^B^, we propose to replace the nonadditive exchange functional by its exact, nonlocal counterpart. The nonadditive exchange correction for SCF-DMRG-in-DFT energy is therefore defined as

20 $$\Delta_{x}^{\mathrm{nadd}}=-E_{x}^{\mathrm{DFT},\mathrm{nadd}}\left[\gamma_{\mathrm{emb}}^{A},\gamma^{B}\right]+E_{\mathrm{xx}}^{\mathrm{nadd}}\left[\gamma_{\mathrm{emb}}^{A},\gamma^{B}\right]$$

The nonadditive exact exchange energy is of the form

21 $$E_{\mathrm{xx}}^{\mathrm{nadd}}\left[\gamma_{\mathrm{emb}}^{A},\gamma^{B}\right]=-\sum_{\sigma }\sum_{\mathrm{pq}\in A}\sum_{\mathrm{rs}\in B}{\left[\gamma_{\mathrm{emb}}^{A}\right]}_{p_{\sigma }q_{\sigma }}{\left[\gamma^{B}\right]}_{r_{\sigma }s_{\sigma }}\left\langle \mathrm{pr}\right|\mathrm{sq}\rangle$$

where A and B denote disjoint subsets of orbitals assigned to the fragments.

To improve the description of the nonadditive correlation energy in DMRG-in-DFT embedding, we propose to recover dynamic nonadditive correlation within the framework of the multireference adiabatic connection approximation.^8,9^ In this approach, the AC expression for the correlation energy associated with a given reference wave function Ψ^ref^ is obtained by linearly interpolating between the zeroth-order Hamiltonian *Ĥ* ^(0)^ and the exact Hamiltonian Ĥ

22 $$\forall_{\alpha \in \left[0,1\right]}{Ĥ}^{\alpha }={Ĥ}^{\left(0\right)}+\alpha {Ĥ}^{\prime },,\mathrm{with},{Ĥ}^{\prime }=Ĥ-{Ĥ}^{\left(0\right)}$$

and the correlation energy formula formally reads

23 $$\begin{array}{l} E_{\mathrm{corr}}^{\mathrm{AC}}=\int_{0}^{1}W^{\alpha }d\alpha \\ W^{\alpha }=\left\langle \Psi^{\alpha }|{Ĥ}^{\prime }|\Psi^{\alpha }\rangle -\langle \Psi^{\mathrm{ref}}|{Ĥ}^{\prime }|\Psi^{\mathrm{ref}}\right\rangle \end{array}$$

where *W* ^α^ is the exact AC integrand and Ψ^α^ denotes the ground state of *H* ^α^, cf. eq 22.

The exact adiabatic connection formulation is turned into an efficient and accurate approximation, denoted AC0, by the following steps: (1) assuming that the one-particle reduced density matrix (1-RDM) remains constant along the AC path; (2) expanding the AC integrand around α = 0 up to linear order in α, noting that *W* ^(0)^ = 0 (i.e., there is no correlation at α = 0), and performing the integration,

24 $$E_{\mathrm{corr}}^{\mathrm{AC}0}=\frac{1}{2}W_{\alpha =0}^{\left(1\right)}$$

and (3) expressing the first-order AC integrand in terms of α-dependent one-electron transition density matrices, which are evaluated within the extended random phase approximation (ERPA)^37^ at first order in α.^8^ Ultimately, the AC0 correlation energy can be written in the compact form

25 $$E_{\mathrm{corr}}^{\mathrm{AC}0}=\sum_{\mathrm{pqrs}}T_{\mathrm{pq},\mathrm{rs}}^{\mathrm{AC}0}\left\langle \mathrm{pr}\right|\mathrm{qs}\rangle$$

where the AC0 correlation amplitudes *T* ^AC0^ are constructed from the ERPA eigenvectors (see, for example, ref 9 for explicit expressions). Importantly, the evaluation of the AC0 correlation energy requires reduced density matrices of the reference wave function of order no higher than two, which constitutes a clear advantage over multireference second-order perturbation theories that rely on higher-order RDMs.^38^ The low computational cost of AC0 does not compromise its accuracy, which is comparable to that of the more expensive PT2 methods.^18,39^

The nonadditive correlation correction, Δ_*c*_^nadd^, is formulated by exploiting the AC0 correlation energy expression and retaining only the terms that describe interfragment correlation

26 $$\Delta_{c}^{\mathrm{nadd}}=-E_{c}^{\mathrm{DFT},\mathrm{nadd}}\left[\gamma_{\mathrm{emb}}^{A},\gamma^{B}\right]+E_{c}^{\mathrm{AC}0,\mathrm{nadd}}\left[\gamma_{\mathrm{emb}}^{A},\Gamma_{\mathrm{emb}}^{A},\gamma^{B}\right]$$

Note that the computation of *E*_*c*_^AC0,nadd^ requires both the embedded 1- and 2-RDMs of the fragment A, γ_emb_^A^ and Γ_emb_^A^, respectively. For compactness, in equations below we indicate the dependence on the 1-RDM only. A physical nonadditive correlation energy must vanish in the limit of dissociation of the composite system into noninteracting fragments. Accordingly, the construction of the nonadditive AC0 correlation energy is subject to a condition

27 $$\lim_{R_{\mathrm{AB}}\to \infty }E_{c}^{\mathrm{AC}0,\mathrm{nadd}}\left[\gamma_{\mathrm{emb}}^{A},\gamma^{B}\right]=0$$

Let us analyze the general AC0 correlation energy expressed in terms of products of the correlation amplitudes *T* ^AC0^ and two-electron integrals,^9,38^ as given in eq 25. If the orbital space is partitioned into active (a), inactive (doubly occupied, o), and virtual (v) orbitals, corresponding to fractional, 2, or 0 occupation numbers, respectively, then only the following combinations of pairs (*pq*) and (*rs*) are allowed: (ao)­(ao), (va)­(va), (vo)­(aa), (va)­(aa), (aa)­(ao), (va)­(ao), (vo)­(ao), (vo)­(vo), (va)­(vo). Here, the notation (vo)­(ao) indicates that in the first pair (*pq*), one orbital is virtual (v) and one is inactive (o), while in the second pair (*rs*), one orbital is active (a) and the other is inactive (o).

A completion of the DMRG-in-DFT calculation leads to obtaining a set of occupied orbitals localized on the subsystem B (those are the frozen KS-DFT orbitals), which will be denoted by o_B_, and a set of active orbitals assigned to the subsystem A. If the concentric localization procedure,^40^ or some alternative truncation scheme, has been adopted to reduce the dimension of the orbital space for DMRG calculation, then there will also be a set of virtual (v) orbitals. However, by construction the virtual orbitals do not correlate with the active orbitals, consequently the *T*_(va)(..)_^AC0^ AC0 amplitudes are assumed to be negligible. We now consider which of the remaining amplitude classes, (ao_B_)­(ao_B_), (vo_B_)­(aa), (aa)­(ao_B_), (vo_B_)­(ao_B_) satisfy condition ([Disp-formula eq27]) and thus must be included in the nonadditive correlation energy. Before analyzing them, one should notice that in the dissociation limit *R*_AB_ → ∞, some active orbitals will localize on the fragment B and their occupation numbers will vanish (they will become uncorrelated). Denote a set of such orbitals as (a_B_). The complementary set, denoted as (a_A_) will consist of the active orbitals which will localize on the subsystem A. Now it is clear that contributions to the AC0 correlation energy from the amplitudes *T*_(ao_B_)(ao_B_)_^AC0^ and *T*_(vo_B_)(ao_B_)_^AC0^ do not vanish in the dissociation limit, as terms with the active orbitals belonging to a subset (a_B_), namely *T*_(a_B_o_B_)(a_B_o_B_)_^AC0^ and *T*_(vo_B_)(a_B_o_B_)_^AC0^, will remain finite (they can be interpreted as those which describe a correlation energy for the subsystem B)

28 $$\exists_{\begin{array}{l} \mathrm{pq}\in \left(a_{B}o\right)\\ \mathrm{rs}\in \left(a_{B}o\right) \end{array}}\wedge \exists_{\begin{array}{l} \mathrm{pq}\in \left(\mathrm{vo}\right)\\ \mathrm{rs}\in \left(a_{B}o\right) \end{array}}\lim_{R_{\mathrm{AB}}\to \infty }T_{\mathrm{pq},\mathrm{rs}}^{\mathrm{AC}0}\neq 0$$

One is therefore left with only two classes of amplitudes, *T*_(vo_B_)(aa)_^AC0^ and *T*_(aa)(ao_B_)_^AC0^. Consider first the case when both active indices in a pair (aa) correspond to orbitals localized on subsystem B. In this situation, the amplitudes vanish in the dissociation limit, as the occupancies of the orbitals a_B_ approach zero, namely

29 $$\forall_{p\in \left(a_{B}\right)}\lim_{R_{\mathrm{AB}}\to \infty }n_{p}=0\Rightarrow \forall_{\begin{array}{l} \mathrm{pq}\in \left(\mathrm{vo}\right)\\ \mathrm{rs}\in \left(a_{B}a_{B}\right) \end{array}}\wedge \forall_{\begin{array}{l} \mathrm{pq}\in \left(a_{B}o\right)\\ \mathrm{rs}\in \left(a_{B}a_{B}\right) \end{array}},\lim_{R_{\mathrm{AB}}\to \infty }T_{\mathrm{pq},\mathrm{rs}}^{\mathrm{AC}0}=0$$

In all other cases, the (vo_B_)­(aa) and (aa)­(ao_B_) amplitudes involve at least one active orbital from a subset (a_A_). Since orbitals o_B_ are localized on B, these amplitudes also vanish in the dissociation limit (recall that only amplitudes with indices corresponding to orbitals localized on the same fragment remain finite). Altogether, we obtain

30 $$\forall_{\begin{array}{l} \mathrm{pq}\in \left({\mathrm{vo}}_{B}\right)\\ \mathrm{rs}\in \left(\mathrm{aa}\right) \end{array}}\wedge \forall_{\begin{array}{l} \mathrm{pq}\in \left({\mathrm{ao}}_{B}\right)\\ \mathrm{rs}\in \left(\mathrm{aa}\right) \end{array}}\lim_{R_{\mathrm{AB}}\to \infty }T_{\mathrm{pq},\mathrm{rs}}^{\mathrm{AC}0}=0$$

The proposed nonadditive AC0 correlation energy for DMRG-in-DFT, which satisfies the condition in (eq 27), includes therefore only two types of amplitudes, namely

31 $$E_{c}^{\mathrm{AC}0,\mathrm{nadd}}=2\sum_{\begin{array}{l} \left(pq\right)\in \left({\mathrm{vo}}_{B}\right)\\ \left(rs\right)\in \left(\mathrm{aa}\right) \end{array}}T_{\mathrm{pq},\mathrm{rs}}^{\mathrm{AC}0}\left\langle \mathrm{pr}|\mathrm{qs}\right\rangle +2\sum_{\begin{array}{l} \left(pq\right)\in \left(\mathrm{aa}\right)\\ \left(rs\right)\in \left({\mathrm{ao}}_{B}\right) \end{array}}T_{\mathrm{pq},\mathrm{rs}}^{\mathrm{AC}0}\left\langle \mathrm{pr}|\mathrm{qs}\right\rangle$$

­(a factor 2 results from exploiting the symmetry *T*_*pq*,*rs*_^AC0^ = *T*_*rs*,*pq*_^AC0^). The total DMRG-in-DFT energy, corrected for the nonadditive exchange and correlation contributions, is finally given by

32 $$E=E_{\mathrm{DMRG}‐\mathrm{in}‐\mathrm{DFT}}+\Delta_{x}^{\mathrm{nadd}}+\Delta_{c}^{\mathrm{nadd}}$$

To reduce the computational cost of treating subsystem A, one may replace DMRG with CASSCF within the embedding framework, thereby restricting the wave function treatment to a substantially smaller active space. In principle, DMRG-in-DFT assumes an active space comprising all electrons assigned to subsystem A together with all orbitals not belonging to the environment. For realistic systems, however, this prescription can lead to very large active spaces that remain prohibitively expensive even when DMRG is employed. In this work, we therefore investigate the performance of CASSCF-in-DFT, with the aim of assessing the trade-off between computational efficiency and accuracy resulting from the use of reduced active spaces. Employing CASSCF instead of DMRG has two main implications. First, unlike DMRG, the CASSCF energy of subsystem A does not include dynamic correlation, which necessitates an additional evaluation of the corresponding correlation energy. Second, in contrast to the DMRG case with concentric localization, all active orbitals in CASSCF remain localized on fragment A and are correlated with the virtual orbitals. Consequently, the nonadditive correlation energy will involve a larger set of AC0 amplitude classes than those considered for DMRG-in-DFT as shown in eq 31.

To formulate a nonadditive correlation energy expression appropriate for CAS-in-DFT, we require, as before, that it satisfies the dissociation condition given in eq 27. Since a given amplitude *T*_*pq*,*rs*_^AC0^ vanishes unless all orbitals *p*, *q*, *r*, *s* are localized on the same fragment in the dissociation limit, and because both inactive and active orbitals used in the CAS wave function remain localized on subsystem A in this limit, it follows that all amplitudes for which at least one index corresponds to subsystem A and at least one to subsystem B vanish upon dissociation. All such amplitudes are included in the computation of the nonadditive correlation energy

33 $$E_{c}^{\mathrm{AC}0,\mathrm{nadd}}=\sum_{\left(\mathrm{pqrs}\right)\in (A-B{)}_{c}}T_{\mathrm{pq},\mathrm{rs}}^{\mathrm{AC}0}\left\langle \mathrm{pr}\right|\mathrm{qs}\rangle$$

The notation (*pqrs*) ∈ (**A** − **B**)_*c*_ indicates that a correlation amplitude *T*_*pq*,*rs*_ belongs to one of the classes listed in the first column of Table 1. For example, if *pqr* pertain to active orbitals (by default localized on A) and *s* denotes a doubly occupied orbital localized on B, then the amplitude is of the (aa)­(ao_B_) type. Since it vanishes in the strict separation limit of the subsystems, lim_*R*_AB_→∞_ *T*_*pq*,*rs*_^AC0^ = 0, it is included in the set (**A** − **B**)_*c*_. In contrast, if *s* pertained to a doubly occupied orbital localized on A, the amplitude would be of the (aa)­(ao_A_) type, which does not vanish in the dissociation limit and is therefore excluded from the summation in eq 33.

Comparing the expressions for the nonadditive correlation energy in CAS-in-DFT and DMRG-in-DFT, one observes that the latter is more restrictive. In particular, a larger number of amplitude classes contributes to the nonadditive correlation in the CAS case. Achieving a nonadditive correlation of comparable magnitude in DMRG-in-DFT would require explicitly distinguishing between active orbitals localized on subsystems A and B in the dissociation limit. With the present definitions, one therefore expects the magnitude of the nonadditive correlation energy to be larger for CAS-in-DFT than for DMRG-in-DFT.

**Table 1:** Partitioning of AC0 Amplitudes into Contributions to Nonadditive Correlation (First Column) and Subsystem A Correlation Energy (Second Column) in CAS-in-DFT^a^

(**A** − **B**)_*c*_	**A**_*c*_
(aa)(ao_B_)*	(aa)(ao_A_)

(aa)(vo_B_)*	(aa)(vo_A_)

(va)(ao_B_)	(va)(ao_A_)

(va)(vo_B_)	(va)(vo_A_)

(ao_B_)(ao_B_)	(ao_A_)(ao_A_)
(ao_B_)(ao_A_)	

(vo_B_)(ao_A_)	(vo_A_)(ao_A_)
(vo_A_)(ao_B_)	
(vo_B_)(ao_B_)	

(vo_A_)(vo_B_)	(vo_A_)(vo_A_)

	(va)(aa)

	(va)(va)

a o_A_ and o_B_ denote inactive orbitals assigned to subsystems A and B, respectively, while a and v pertain to active (fractionally occupied, assigned to A) and virtual orbitals. For the DMRG-in-DFT variant only two classes of terms contribute to the nonadditive correlation energy; these are marked with an asterisk.

Amplitudes *T*_*pq*,*rs*_^AC0^ contributing to the dynamic correlation energy of subsystem A are those for which none of the indices *p*,*q*,*r*,*s* correspond to orbitals assigned to subsystem B. The total CAS-in-DFT energy, corrected for the correlation energy of subsystem A as well as the nonadditive exchange and correlation contributions, is therefore given by

34 $$E=E_{\mathrm{CAS}‐\mathrm{in}‐\mathrm{DFT}}+\sum_{\left(\mathrm{pqrs}\right)\in A_{c}}T_{\mathrm{pq},\mathrm{rs}}^{\mathrm{AC}0}\left\langle \mathrm{pr}\right|\mathrm{qs}\rangle +\Delta_{x}^{\mathrm{nadd}}+\Delta_{c}^{\mathrm{nadd}}$$

where the summation runs over all AC0 amplitudes associated with the correlation on subsystem A, i.e., those which belong to a set **A**_*c*_ presented in the second column of Table 1.

The proposed corrections to the nonadditive correlation energy are based on the AC0 method, as it provides an efficient ab initio route for computing multireference correlation energies in large active spaces, making it particularly suitable for DMRG embedding calculations. Nevertheless, an analogous nonadditive correlation correction could equally well be formulated using a multireference perturbation theory method, such as CASPT2 ^6^ or NEVPT2,^7^ by following the same principles as in AC0 [see eqs 28–30] to select the PT2 amplitudes,^41^ *T*_*pqrs*_^PT2^, contributing to the nonadditive correlation energy *E*_c_^PT2,nadd^.

Finally, we note that the exchange and correlation corrections proposed in this work represent multireference extensions of the analogous corrections introduced by Goodpaster et al.^24^ to reduce the CCSD­(T)-in-DFT embedding error. In particular, for a single-reference description of the active fragment A, the AC0 amplitudes appearing in eqs 31 and 34 reduce to the MP2 amplitudes *T*_*ia*,*jb*_^MP2^, where *i* ∈ A, *j* ∈ B, and *a*,*b* denote virtual orbitals. In this limit, the nonadditive correlation correction becomes identical to that proposed in ref 24. The nonadditive exchange energy correction introduced here, cf. eq 20, is formally analogous to that of Goodpaster et al., with the difference that in the present work a correlated density matrix of subsystem A is employed, whereas in ref 24 the uncorrelated (Hartree−Fock) embedded density matrix was used.

## 3 Computational Details

We applied the methodology described in the previous section to two prototypical molecular systems containing strongly correlated fragments. As a representative example, we considered a linear chain of 20 hydrogen atoms (H_20_, Figure 1a), with the central active fragment consisting of four equally spaced hydrogen atoms. To study the effect of increasing multireference character, we varied the interatomic distances within this fragment from 0.7 to 2.5 Å. The remaining hydrogen atoms form the environment, arranged as hydrogen dimers with an intradimer bond length of 1.0 Å and an interdimer separation of 1.4 Å. The latter distance also corresponds to the separation between the hydrogen nuclei at the edges of subsystem A and the nearest hydrogen nuclei belonging to the environment. The distance 1.4 Å is short enough so that covalent bonds between the central H_4_ unit and the neighboring hydrogen atoms are not fully broken. As discussed below, this distance ensures strong coupling between the edge atoms of fragment A and those of fragment B. To assess the effect of enlarging the active fragment, we also examined a case where the active region contains eight hydrogen atoms, the four stretched central atoms plus the two nearest hydrogen dimers (one on each side). The hydrogen chain was chosen for its one-dimensional character, as the DMRG method can provide reliable benchmark energies of near-FCI quality for both basis sets employed, namely 6−31G^42^ and cc-pVDZ.^43^

**Figure 1:**
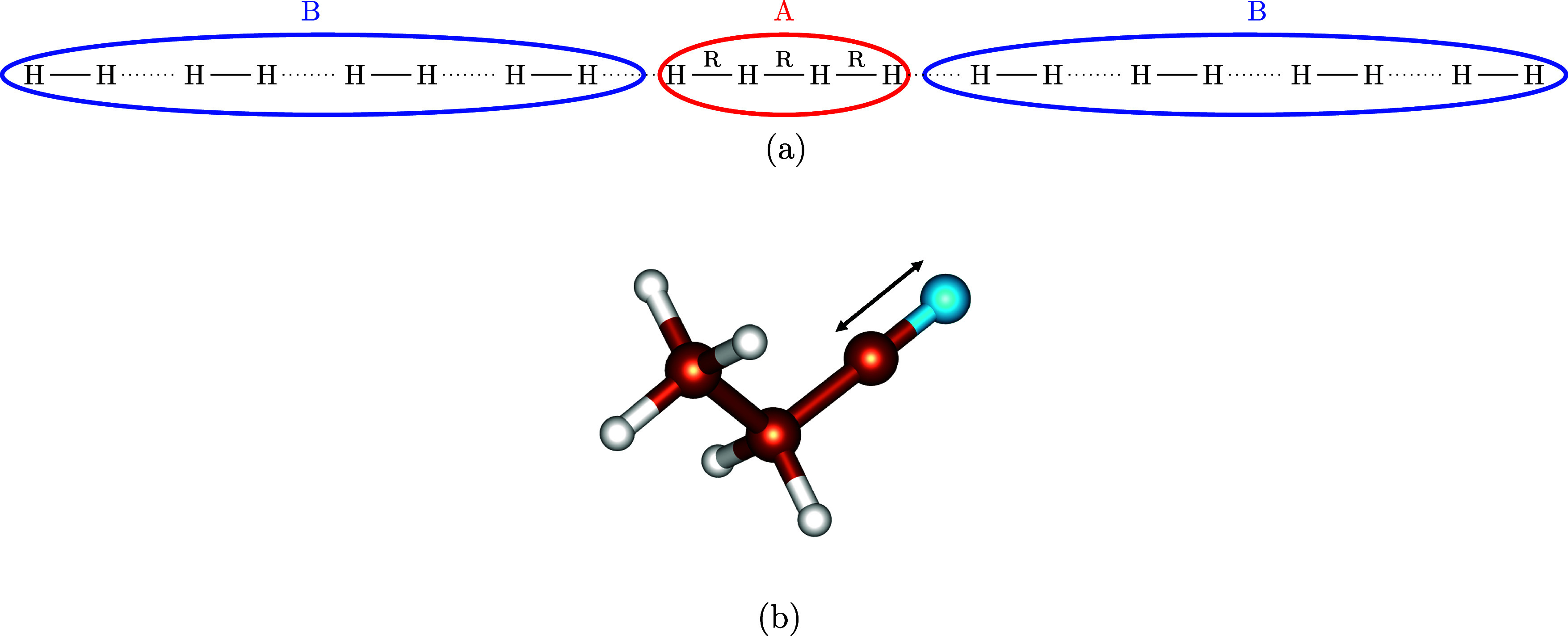
Benchmark problems investigated in this work: (a) H_20_ chain with the central active fragment (A) composed of four hydrogen atoms, where the interatomic distances (*R*) are varied, (b) triple bond stretching in propionitrile (CH_3_CH_2_CN) with the active fragment defined as the −CN group.

The second system we investigated is propionitrile (CH_3_CH_2_CN, Figure 1b), for which we adopted the molecular geometry from our previous work on DMRG-in-DFT.^31^ The active subsystem was defined as the −CN group, and by stretching the C≡N triple bond from 0.85 to 2.5 Å, we modulated its multireference character. In the case of propionitrile, all calculations were performed using the cc-pVDZ basis set.^43^ As a reference, we used DMRG energies within the frozen-core approximation, taken from ref 31.

In the following section, we compare two WF-in-DFT approaches: DMRG-in-DFT^31^ and CASSCF-in-DFT,^32^ which we denote shortly as CAS-in-DFT. The occupied orbitals, obtained from the KS-DFT computation on the entire molecule, were partitioned into active (A) and environment (B) subspaces using the SPADE procedure.^44^ For DMRG-in-DFT, a preliminary HF-in-DFT calculation was performed. In order to maintain orthogonality between the active and environment orbitals, we employed the parameter-free approach diagonalizing the modified Fock matrix with the environment degrees of freedom projected out.^25^ In the case of CASSCF-in-DFT, the original projector method^23^ was applied with a projector scaling parameter of 10^6^, as this approach is technically straightforward to implement. Additionally, for DMRG-in-DFT, we employed the concentric localization (CL) technique^40^ to reduce the size of the virtual space.

All DMRG calculations, both embedded and reference, were carried out in a basis of Pipek−Mezey^45^ split-localized molecular orbitals^46^ by means of the MOLMPS program.^47^ Orbital ordering was optimized using the Fiedler method^48^ applied to the matrix of exchange integrals,^46^ and the calculations were initialized with the CI-DEAS procedure.^14,49^ We employed the dynamical block state selection (DBSS) scheme,^50^ which adapted the bond dimension to achieve a target truncation error of 10^−6^. The minimum bond dimension and the fixed bond dimension used during the first warm-up sweep were both set to 500 for the embedded calculations and 1000 for the reference ones.

The CASSCF as well as DFT calculations were performed with ORCA package.^51^ In both systems, the active space was defined as the strongly correlated orbital subspace of the active fragment, CAS­(4,4) and CAS­(8,8) for H_20_ with four- and eight hydrogen atoms in the active fragment, respectively, and CAS­(6,6) for propionitrile. The projection-based WF-in-DFT protocol was implemented in a local version of ORCA 5.0. For DFT calculations, we employed the B3LYP,^52–54^ and PBE^55^ exchange−correlation functionals.

The AC0 correlation energy corrections were computed in the GammCor program.^56^ The amplitudes listed in Table 1 were obtained from the general expression given, for example, in eq (79) of ref 38. In general, computing the AC0 amplitudes requires solving the zeroth-order extended random phase approximation (ERPA) equations,^37^ which involve an uncorrelated Hamiltonian *Ĥ* ^(0)^ constructed for a given reference wave function. For the embedding schemes considered here, the one-electron part of *Ĥ* ^(0)^ must be modified to reflect that the active orbitals in DMRG are optimized in the presence of the embedding potential generated by the environment, whereas in CASSCF they are influenced by both the embedding potential and the inactive orbitals localized on subsystem A. The explicit forms of the effective one-electron Hamiltonians employed for evaluating the AC0 amplitudes, and consequently the corresponding nonadditive correlation energy corrections in the DMRG-in-DFT and CAS-in-DFT schemes, are provided in the Appendix.

## 4 Results and Discussion

### 4.1 H_20_ Chain

We begin by comparing a standard one-shot WF-in-DFT calculation to a self-consistent (SCF) WF-in-DFT scheme. In the one-shot variant, the embedding potential is built from the initial DFT density, while in the SCF variant, the WF-in-DFT calculations are run repeatedly as macro-iterations and the embedding potential is updated at each macro-iteration using the actual WF density. The latter mitigates so-called density-driven errors,^57^ which arise when the initial DFT density does not adequately describe the active subsystem. This comparison was carried out for DMRG-in-B3LYP, without truncation of the virtual space in the DMRG calculation of the active fragment, on the H_20_ chain in the 6−31G basis with a four-atom active fragment. The results are shown in Figure 2. As expected, the SCF procedure yields slightly more accurate energies, however, the maximum deviation from the one-shot result is only about 1 kcal/mol. In practice, the perturbative “trace” term in eq 1 performs very well. We note that the one-shot approach becomes insufficient for excited states, when the embedding potential is constructed from a ground-state DFT description.

**Figure 2:**
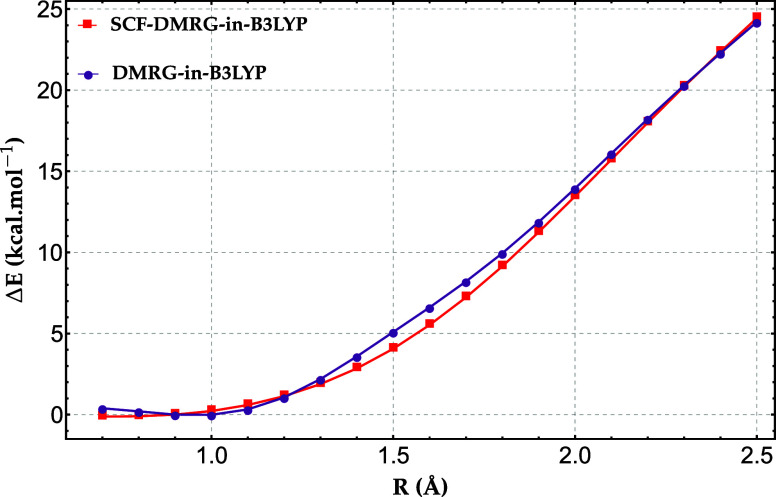
Relative energy error (kcal·mol^−1^) for the H_20_ chain comparing self-consistent DMRG-in-B3LYP and standard DMRG-in-B3LYP, using a four-atom active fragment. Errors are referenced to full-system DMRG energies with the 6−31G basis set.

In Figure 3, we illustrate the performance of concentric localization for the H_20_ chain with 4 atoms in the active fragment using the 6−31G basis. Remarkably, subkcal/mol accuracy is already achieved with the first CL shell (*n* = 0), corresponding to carrying out a DMRG calculation for 4 electrons in a set of 10 orbitals, which corresponds to an active space of CAS­(4,10). This is significantly smaller than the untruncated active space, CAS­(4,32). Including a second CL shell (*n* = 1) fully reproduces the nontruncated results.

**Figure 3:**
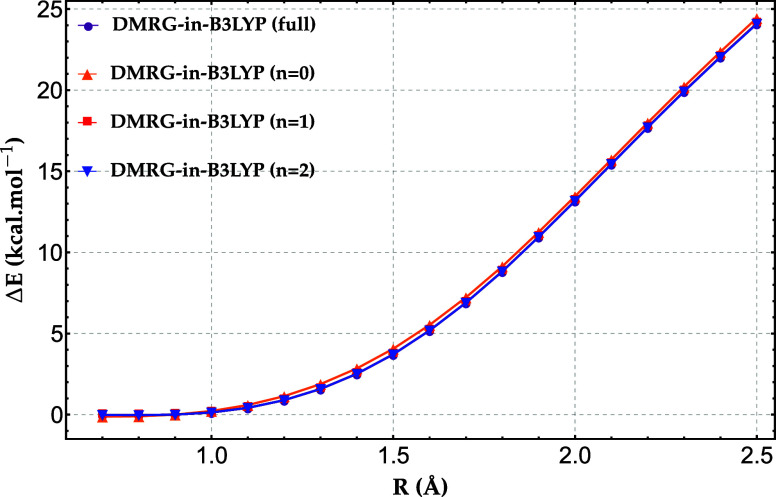
Relative energy error (in kcal/mol) of DMRG-in-B3LYP for the H_20_ chain with 4 atoms in the active fragment, compared to full-system DMRG, using the 6−31G basis set. The labels “full”, *n* = 0, *n* = 1, and *n* = 2 denote the calculations without concentric localization, and with the first, second, and third CL shells, respectively.

Based on the observations in the 6−31G basis, we employed the one-shell CL approximation for subsequent calculations in the larger cc-pVDZ basis. Moreover, to eliminate any possible density-driven errors, we employ the SCF-WF-in-DFT approach (the abbreviation SCF is not explicitly specified from now on).

We now assess the accuracy of DMRG-in-B3LYP/cc-pVDZ, both without and with nonadditive exchange−correlation corrections, for the H_20_ cases with 4 atoms in the active fragment (Figure 4a) and 8 atoms in the active fragment (Figure 4b). As shown in Figure 4a, when the active fragment is restricted to four atoms, the energetic error of the parent DMRG-in-B3LYP method rises to nearly 25 kcal/mol for the most stretched bonds, reflecting the well-known tendency of approximate DFT functionals to accumulate large static correlation errors in strongly correlated systems. As shown in Figure S1 in the Supporting Information (SI), the errors in the total energies obtained with bare B3LYP and PBE functionals exceed 50 and 35 kcal/mol, respectively, in the dissociation limit of the central H_4_ fragment. This large static-correlation error in the hydrogen chain can be primarily attributed to the fractional-spin error,^58^ as confirmed by Figure S4. The latter shows that removing the fractional-spin error from the PBE relative energies of the entire hydrogen chain yields a dissociation curve in excellent agreement with the reference data. Most of this error arises from the exchange functional, while the correlation contribution accounts for only about 20% of the total error.

**Figure 4:**
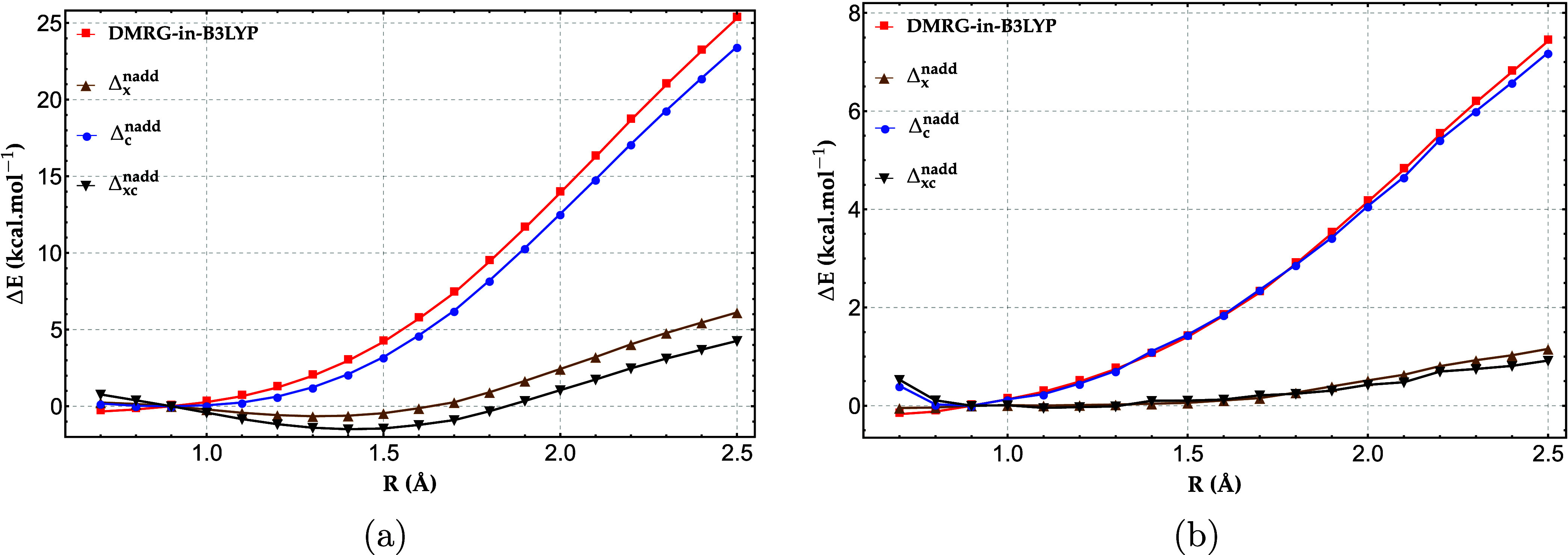
Relative energy errors (in kcal/mol) of DMRG-in-B3LYP and DMRG-in-B3LYP with the nonadditive exchange (Δ_*x*_^nadd^), correlation (Δ_*c*_^nadd^), and exchange−correlation (Δ_*xc*_^nadd^) corrections, for H_20_ chain with (a) 4-atom active fragment and (b) 8-atom active fragment, benchmarked against full-system DMRG with the cc-pVDZ basis set.

The error extends over the central H_4_ fragment and even beyond the first pair of hydrogen dimers adjacent to it. This indicates that correcting the static-correlation error solely within the H_4_ or even H_8_ fragment, by treating it with DMRG as in DMRG-in-DFT, does not suffice to recover accurate total energies for the full system. In other words, in the systems studied here, fragment A (whether H_4_ or H_8_) remains strongly coupled to its environment, and the DFT treatment of the nonadditive exchange−correlation energy fails to account for this coupling.

Incorporating the nonadditive exact exchange together with the AC0 correlation correction significantly suppresses the error of the *E*_*xc*_^nadd^ energy, reducing it to about 4 kcal/mol, with the improvement being dominated by the exchange contribution. This is consistent with the observation, cf. Figure S4 in SI, that the contribution to the total fractional spin error from the correlation functional is almost an order of magnitude smaller than that of the exchange functional (for details, see SI). Increasing the size of the active fragment by including one adjacent hydrogen dimer on each side of the H_4_ chain in subsystem A reduces the coupling between the subsystems, as most of the static-correlation effects are now captured within A. Consequently, as shown in Figure 4b, the maximum error in the DMRG-in-B3LYP relative energy is much smaller than in the previous case, amounting to less than 8 kcal/mol. With the nonadditive exchange−correlation correction it drops further to less than 1 kcal/mol, here almost entirely due to the exchange term as the magnitude of the nonadditive correlation energy is much smaller. In SI we show that DMRG-in-PBE is as accurate as the B3LYP-based embedding (see Figures S2 and S3), which indicates that hybrids may in practice be replaced with a less expensive, pure GGA model.

Since DMRG-in-DFT, even with CL, can become prohibitively expensive for larger systems due to the size of the virtual space, we also examined the performance of CAS-in-DFT. Figure 5 shows CAS-in-B3LYP/cc-pVDZ results for the H_20_ chain with (a) four atoms and (b) eight atoms in the active fragment. While CAS-in-B3LYP provides a reasonable description, it lacks dynamic electron correlation, which is often essential for achieving chemical accuracy. A comparison of the maximal errors obtained with CAS-in-DFT and DMRG-in-DFT for the case of a four-atom active fragment may give the false impression that CAS-in-DFT is more accurate. In fact, this apparent improvement arises from a fortuitous cancellation between the negative error in the CASSCF relative energy, caused by the missing dynamic correlation, and the positive error in the nonadditive exchange−correlation contribution. When the active fragment is enlarged to eight atoms, this favorable cancellation disappears, and the CAS-in-DFT results become less accurate than those of DMRG-in-DFT. Hence, achieving consistent accuracy within CAS-in-DFT requires simultaneous correction of both the CAS and nonadditive exchange−correlation energies. To address this, we applied the AC0 correction to first recover the missing dynamical correlation within the active subsystem. We denote this method as AC0­(A)-CAS-in-B3LYP. The largest errors for CAS-in-B3LYP in the four-atom case occur at intermediate bond stretches. Once the AC0 correction for the A fragment is included, the resulting error profile changes, see AC0­(A)-CAS­(4,4)-in-B3LYP in Figure 5a, and closely resembles that of DMRG-in-B3LYP with the largest errors for the most stretched bonds. This picture is consistent with the fact that AC0 captures the dominant part of dynamic electron correlation, thereby shifting the CAS-in-B3LYP results toward DMRG-in-B3LYP. A similar effect is observed for the eight-atom active fragment: describing it solely at the CASSCF level leads to a substantially negative error in the relative energy, whereas inclusion of dynamical correlation for the active fragment brings the CAS-in-B3LYP results into close agreement with DMRG-in-B3LYP.

**Figure 5:**
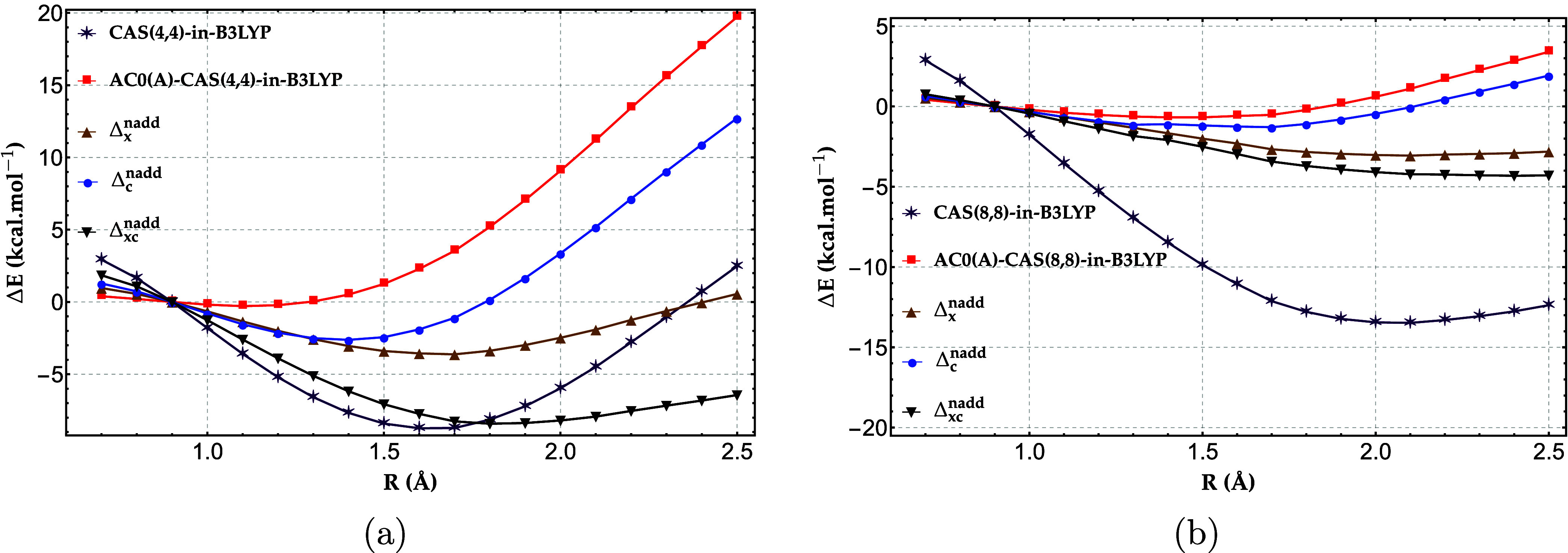
Relative energy errors (in kcal/mol) of CAS-in-B3LYP, AC0­(A)-CAS-in-B3LYP, and AC0­(A)-CAS-in-B3LYP with the nonadditive exchange (Δ_*x*_^nadd^), correlation (Δ_*c*_^nadd^), and exchange−correlation (Δ_*xc*_^nadd^) corrections, for H_20_ chain with (a) 4-atom active fragment and (b) 8-atom active fragment, benchmarked against full-system DMRG with the cc-pVDZ basis set.

Next, we focus on assessing the impact of the nonadditive exchange−correlation corrections in the case of CAS-in-B3LYP. Adding the nonadditive exchange energy correction to AC0­(A)-CAS-in-B3LYP results in the same effect as in DMRG-in-B3LYP, reducing the maximal relative energy error by 20 and 7 kcal/mol for the four- and eight-atom active fragment partitions, respectively.

As presented in the Theory section, the nonadditive correlation energy correction for CAS-in-DFT is derived under the same assumption as for DMRG-in-DFT—namely, that only the AC0 amplitudes which vanish in the limit of infinite separation between the active fragment and the environment are retained in the correlation energy expression. Nevertheless, the numerical values of the resulting corrections are not expected to coincide for the two methods. The first important observation is that the chosen active space in CAS-in-DFT consists of orbitals exclusively localized on the active fragment in the separation limit. This has important implications for the contributions to the nonadditive correlation energy, as compared to the DMRG-in-DFT case. Since no active orbitals will be localized on the environment fragment when the fragments are infinitely separated, we cannot apply the same exclusions as in the latter method. In particular, the terms containing AC0 amplitudes *T*_(ao)(ao)_^AC0^ and *T*_(vo)(ao)_^AC0^, satisfy eq 27 and must now be retained in the nonadditive correlation energy formula. In fact, all terms that mix indices from the occupied (o), active (a), and virtual (v) spaces contribute to the latter. Another difference arises from the treatment of the virtual space. Unlike in DMRG-in-DFT, in CAS-in-DFT we do not employ the CL and truncation of the virtual space. As a consequence, the virtual orbitals do correlate with the active orbitals and *T*_(va)(..)_^AC0^ AC0 amplitudes remain finite, which further enhances the overall contribution of the nonadditive correlation energy. This trend is clearly reflected in the results presented in Figure 5a,5b. The inclusion of the nonadditive correlation correction yields a noticeable improvement in AC0­(A)-CAS-in-B3LYP. However, adding the nonadditive exchange contribution on top leads to an underestimation of the relative energy. Comparable accuracy is obtained for four- and eight-atom active fragments, with errors at the largest bond elongations near −5 kcal/mol.

### 4.2 Propionitrile

The second system we examined is the triple-bond stretching in propionitrile. The corresponding results are presented in Figures 6 and 7 for DMRG-in-B3LYP/cc-pVDZ and CAS-in-B3LYP/cc-pVDZ, respectively. In the case of DMRG-in-B3LYP, we observe a similar trend to that found for the H_20_ chain: the nonadditive exchange correction has a stronger influence than the nonadditive correlation correction. However, its overall effect is smaller than in the hydrogen chain, resulting in a larger residual error in the corrected DMRG-in-B3LYP energies. For the most stretched bond, the remaining error exceeds 10 kcal/mol, which nevertheless represents a substantial improvement, by more than 10 kcal/mol, relative to the uncorrected DMRG-in-DFT results.

**Figure 6:**
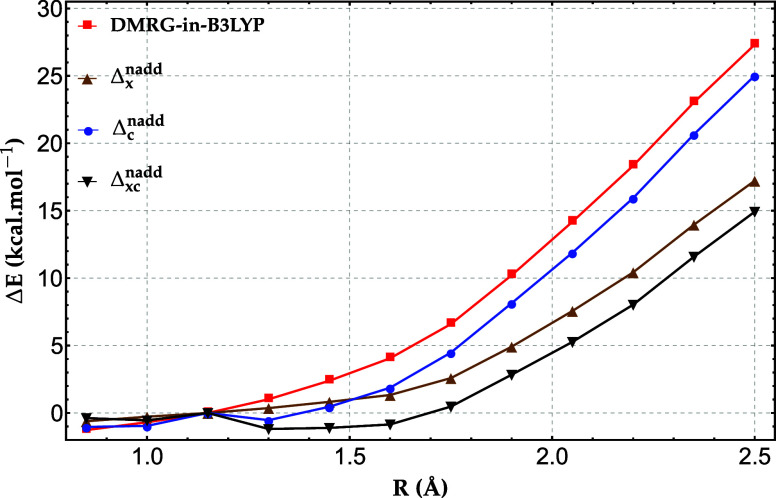
Relative energy errors (in kcal/mol) of DMRG-in-B3LYP and DMRG-in-B3LYP with the nonadditive exchange (Δ_*x*_^nadd^), correlation (Δ_*c*_^nadd^), and exchange−correlation (Δ_*xc*_^nadd^) corrections, for the triple bond stretching in propionitrile (CH_3_CH_2_CN) benchmarked against full-system frozen-core DMRG with the cc-pVDZ basis set.

**Figure 7:**
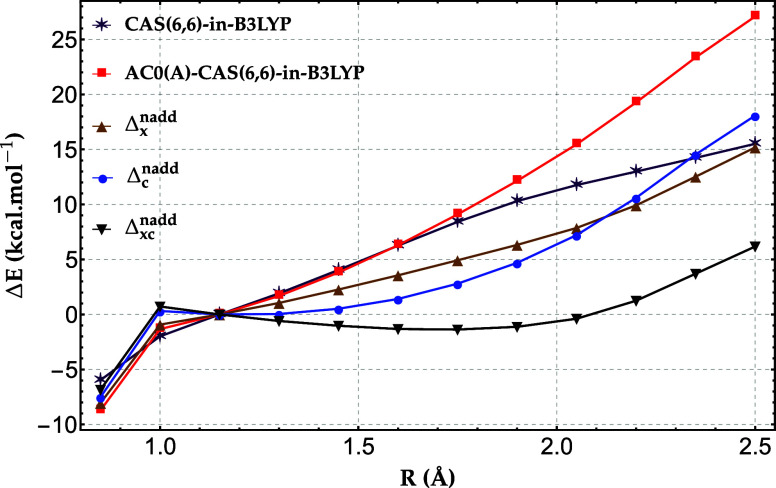
Relative energy errors (in kcal/mol) of CAS­(6,6)-in-B3LYP, AC0­(A)-CAS­(6,6)-in-B3LYP, and AC0­(A)-CAS­(6,6)-in-B3LYP with the nonadditive exchange (Δ_*x*_^nadd^), correlation (Δ_*c*_^nadd^), and exchange−correlation (Δ_*xc*_^nadd^) corrections, for the triple bond stretching in propionitrile (CH_3_CH_2_CN) benchmarked against full-system frozen-core DMRG with the cc-pVDZ basis set.

By contrast, the corrected AC0­(A)-CAS­(6,6)-in-B3LYP results remain within ±5 kcal/mol for most bond lengths, except for the first and last points. As in the H_20_ chain, the uncorrected CAS­(6,6)-in-B3LYP performs slightly better than AC0­(A)-CAS­(6,6)-in-B3LYP due to error cancellation. We also find that the nonadditive correlation correction plays a more prominent role in AC0­(A)-CAS­(6,6)-in-B3LYP than in DMRG-in-B3LYP, accounting for the overall lower errors of the former method compared to the latter. This behavior can be attributed to the fact that the nonadditive correlation term in CAS-in-DFT contains a larger number of contributing amplitudes (cf. Table 1) than its counterpart in DMRG-in-DFT.

## 5 Conclusions

The projection-based DMRG-in-DFT method offers a promising framework for systems in which strong correlation effects are present and localized within a relatively small region of the total system, allowing the DFT description to be replaced by that of a multireference wave function method. However, including all atoms that contribute significantly to static correlation would require a prohibitively large active space, so in practice the wave function fragment remains strongly coupled to its environment. As demonstrated in ref 31, the coupling gives rise to substantial errors in DMRG-in-DFT embedding. In this work, we corroborated this finding by reformulating the total energy expression of the composite system to isolate the nonadditive exchange−correlation energy term, which allowed us to identify it as the principal source of error. To overcome the inability of standard DFT functionals to capture the strong coupling between the active fragment and its environment, we introduced physically motivated corrections for the nonadditive exchange and correlation energies. Specifically, the nonadditive exchange energy is evaluated using the exact exchange functional, while the nonadditive correlation energy is derived from the AC0 correlation energy, retaining only those contributions that vanish in the limit of infinite separation between the fragment and its environment. The new approach enables accurate predictions even with relatively small active fragments.

To demonstrate the reliability of the proposed corrections, we chose two model systems for which DFT fails due to the pronounced static correlation error: a linear H_20_ chain with three central covalent bonds simultaneously stretched, and the propionitrile molecule undergoing dissociation of the C≡N triple bond. While the uncorrected DMRG-in-DFT approach already offers a substantial improvement over pure DFT, the relative energy errors remain considerable—exceeding 25 kcal/mol for the hydrogen chain with a four-atom active fragment and for propionitrile. Upon applying the exchange−correlation energy corrections, the error is significantly reduced in all studied systems.

In practical applications of the projection based DMRG-in-DFT embedding to systems with large environments, evaluating the nonadditive AC0 correlation correction may become prohibitively expensive because of the large number of inactive orbitals assigned to the environment and the extensive virtual space involved (the computational cost of AC0 scales with the fifth power of the number of orbitals). In such cases, one can take advantage of the locality of the environmental orbitals and truncate the ERPA matrices used to compute the AC0 correlation amplitudes, retaining only those orbitals that lie in close proximity to the active fragment. Similar truncation strategy could be adopted for the virtual orbitals upon their localization. An even more efficient alternative could be to employ fractional-spin-error-free functionals for evaluating the nonadditive exchange−correlation energy. Work in these directions is currently in progress.

## Supplementary Material



## Appendix

The computation of AC0 amplitudes for the nonadditive correlation energy follows the procedure outlined in refs 9,38 , with one essential modification: the effective one-electron Hamiltonian, *ĥ* ^(0)^, employed in the zeroth-order ERPA equations must include the embedding potential within the active orbital subspace.

Specifically, if the embedded Ψ^A^ wavefunction follows from DMRG calculation, i.e., all orbitals assigned to A are active, then the effective Hamiltonian is constructed as follows

35
$$h_{\mathrm{pq}}^{\left(0\right)}=\left\{ \begin{array}{ll} h_{\mathrm{pq}}+\upsilon_{\mathrm{pq}}^{\mathrm{emb}} & p,q\in a\\ h_{\mathrm{pq}}+{\left[\mathrm{JK}\right]}_{\mathrm{pq}} & p,q\in o_{B}\;\mathrm{or}\;p,q\in v\\ 0 & \mathrm{otherwise} \end{array}\right.$$

where o_B_ denotes a set of the inactive orbitals assigned to B, v stands to a set of virtual orbitals, which is not empty if DMRG employs truncated space of the HF virtual orbitals. The Coulomb-exchange interaction energy matrix [JK] is defined as follows

36
$$\forall_{\mathrm{pq}\in o_{B}}{\left[\mathrm{JK}\right]}_{\mathrm{pq}}=\sum_{\mathrm{rs}\in a}{\left[\gamma_{\mathrm{emb}}^{A}\right]}_{\mathrm{rs}}\left(\left\langle \mathrm{pr}|\mathrm{qs}\right\rangle -\left\langle \mathrm{pr}|\mathrm{sq}\right\rangle \right)$$

37
$$\forall_{\mathrm{pq}\in v}{\left[\mathrm{JK}\right]}_{\mathrm{pq}}=\sum_{\mathrm{rs}\in a}{\left[\gamma_{\mathrm{emb}}^{A}\right]}_{\mathrm{rs}}\left(\left\langle \mathrm{pr}|\mathrm{qs}\right\rangle -\left\langle \mathrm{pr}|\mathrm{sq}\right\rangle \right)+\sum_{\mathrm{rs}\in o_{B}}{\left[\gamma^{B}\right]}_{\mathrm{rs}}\left(\left\langle \mathrm{pr}|\mathrm{qs}\right\rangle -\left\langle \mathrm{pr}|\mathrm{sq}\right\rangle \right)$$

In the CAS-in-DFT embedding method, the nonadditive correlation energy correction is derived from the AC0 amplitudes obtained using an effective Hamiltonian that accounts for the presence of the inactive orbital set o_B_ localized on subsystem A and reads

38
$$h_{\mathrm{pq}}^{\left(0\right)}=\left\{ \begin{array}{ll} h_{\mathrm{pq}}+{\left[\mathrm{JK}\right]}_{\mathrm{pq}}+\upsilon_{\mathrm{pq}}^{\mathrm{emb}} & p,q\in a\\ h_{\mathrm{pq}}+{\left[\mathrm{JK}\right]}_{\mathrm{pq}} & p,q\in o_{A}\;\mathrm{or}\;p,q\in o_{B}\;\mathrm{or}\;p,q\in v\\ 0 & \mathrm{otherwise} \end{array}\right.$$

where

39
$$\forall_{p,q\in a}{\left[\mathrm{JK}\right]}_{\mathrm{pq}}=\sum_{\mathrm{rs}\in o_{A}}{\left[\gamma_{\mathrm{emb}}^{A}\right]}_{\mathrm{rs}}\left(\left\langle \mathrm{pr}|\mathrm{qs}\right\rangle -\left\langle \mathrm{pr}|\mathrm{sq}\right\rangle \right)$$

40
$$\forall_{p,q\in o_{A}}{\left[\mathrm{JK}\right]}_{\mathrm{pq}}=\sum_{\mathrm{rs}\in a}{\left[\gamma_{\mathrm{emb}}^{A}\right]}_{\mathrm{rs}}\left(\left\langle \mathrm{pr}|\mathrm{qs}\right\rangle -\left\langle \mathrm{pr}|\mathrm{sq}\right\rangle \right)$$

41
$$\begin{array}{l} \forall_{\mathrm{pq}\in o_{B}}{\left[\mathrm{JK}\right]}_{\mathrm{pq}}=\sum_{\mathrm{rs}\in a}{\left[\gamma_{\mathrm{emb}}^{A}\right]}_{\mathrm{rs}}\left(\left\langle \mathrm{pr}|\mathrm{qs}\right\rangle -\left\langle \mathrm{pr}|\mathrm{sq}\right\rangle \right)+\sum_{\mathrm{rs}\in o_{A}}{\left[\gamma_{\mathrm{emb}}^{A}\right]}_{\mathrm{rs}}\left(\left\langle \mathrm{pr}|\mathrm{qs}\right\rangle -\left\langle \mathrm{pr}|\mathrm{sq}\right\rangle \right) \end{array}$$

and the matrix [JK]_*pq*∈v_ is defined in eq 37.

## Notes

Full KS-DFT (with B3LYP and PBE functionals) curves for the H_20_ hydrogen chain and propionitrile (CH_3_CH_2_CN) molecule; DMRG-in-PBE for 4-atom and 8-atom active fragment for the H_20_ hydrogen chain; absolute energies of the H_20_ hydrogen chain for full KS-PBE, DMRG-in-B3LYP, CAS-in-B3LYP and full DMRG; full KS-PBE curve and dissociation energy corrected for the fractional spin error for the H_20_ hydrogen chain; equilibrium geometry and absolute energies for KS-B3LYP, DMRG-in-B3LYP, CAS-in-B3LYP and full DMRG for the propionitrile molecule (PDF)
