# Supplementary material for: Projection-Based DMRG-in-DFT Embedding Corrected by Nonadditive Exchange-Correlation
Source: J Chem Theory Comput. 2026 Feb 9;22(4):1780–92. doi: 10.1021/acs.jctc.5c01930 (PMC13210680; doi:10.1021/acs.jctc.5c01930)
Supplement: Supplementary file 1 [file ct5c01930_si_001.pdf]

Supplementary Information: Projection-based DMRG-in-DFT embedding corrected by nonadditive exchange-correlation

Enzo Monino,[†,∥] Daria Drwal,[‡,∥] Pavel Beran,[†,¶] Michał Hapka,[§] Libor Veis,[*,†] and Katarzyna Pernal[*,‡]

†*J. Heyrovský Institute of Physical Chemistry, Academy of Sciences of the Czech Republic, v.v.i., Dolejškova 3, 18223 Prague 8, Czech Republic*

‡*Institute of Physics, Lodz University of Technology, ul. Wolczanska 217/221, 93-005 Lodz, Poland*

¶*Faculty of Mathematics and Physics, Charles University, 12116 Prague, Czech Republic*

§*University of Warsaw, Faculty of Chemistry, ul. L. Pasteura 1, 02-093 Warsaw, Poland*

∥*Contributed equally.*

E-mail: libor.veis@jh-inst.cas.cz; pernalk@gmail.com

Hydrogen chain

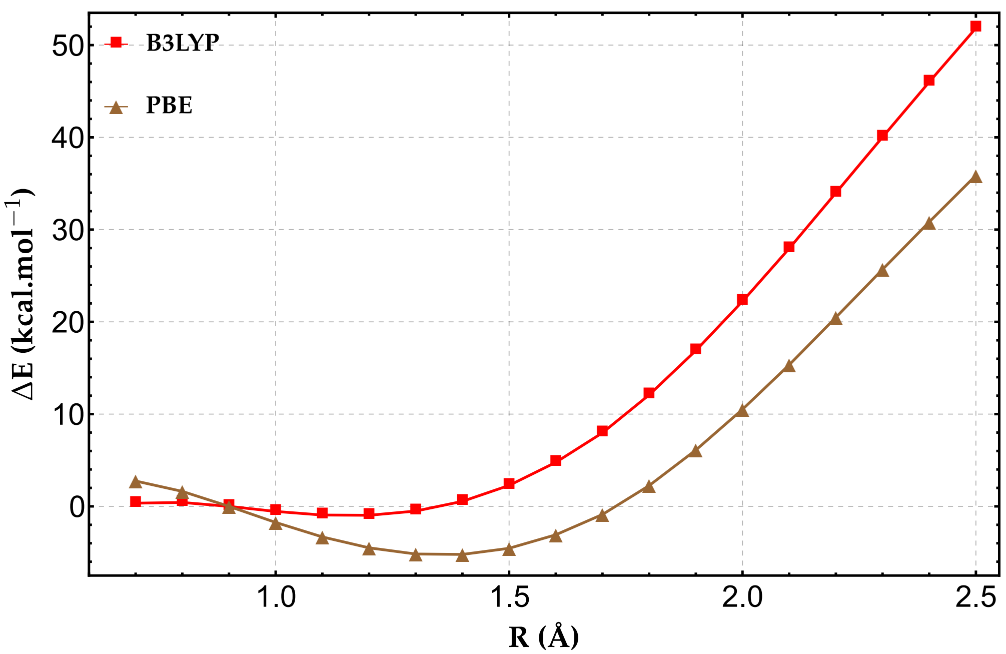

Fig. S1: Relative energy error (kcal·mol^{-1}) for the H_{20} chain comparing B3LYP and PBE functionals. Errors are referenced to full-system DMRG energies with the cc-pVDZ basis set.

Fractional spin error for the H_{20} hydrogen chain

Consider a dissociation limit of the H_{20} chain with the three interatomic distances, R, approaching infinity. The dissociation products are: two hydrogen atoms and two H_9 chains. Since we consider a hydrogen chain in a singlet state, the exchange-correlation energy in this limit, E_{xc}^{∞}, is given by a sum

$$E_{xc}^{\infty} = 2E_{xc}[\rho_\alpha(\mathrm{H}), \rho_\beta(\mathrm{H})] + 2E_{xc}[\rho_\alpha(\mathrm{H}_9), \rho_\beta(\mathrm{H}_9)] \quad (1)$$

where the α and β spin densities localised on a dissociation product X,where X=H or H_9, are equal

$$\rho_\alpha(\mathrm{X}) = \rho_\beta(\mathrm{X}) \quad (2)$$

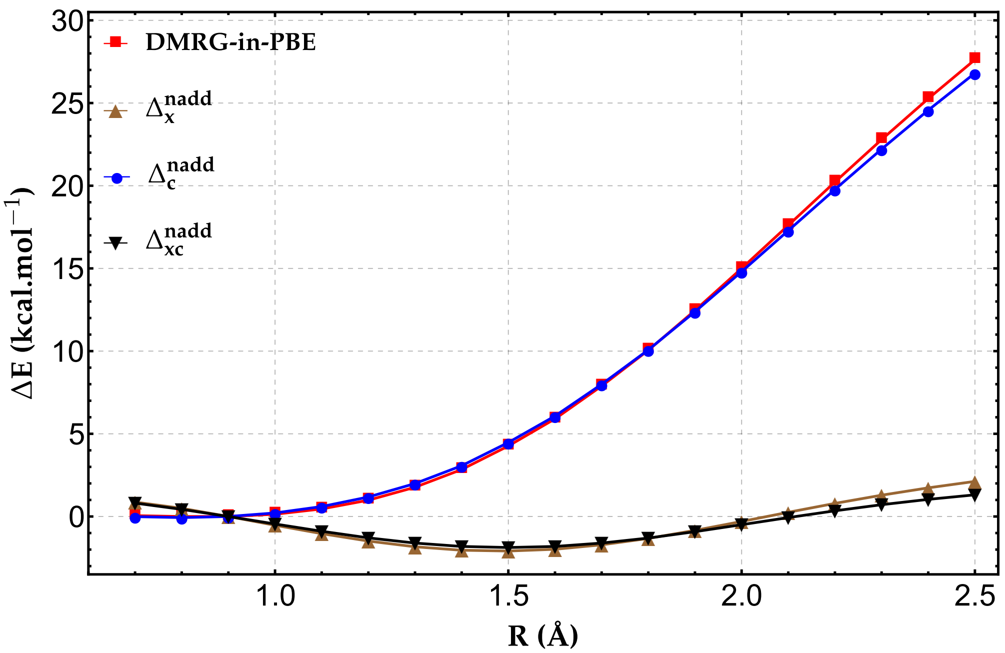

Fig. S2: Relative energy errors (in kcal/mol) of DMRG-in-PBE and DMRG-in-PBE with the nonadditive exchange (Δ_x^{nadd}), correlation (Δ_c^{nadd}), and exchange–correlation (Δ_{xc}^{nadd}) corrections, for 4-atom active fragment for the H_{20} hydrogen chain, benchmarked against full-system DMRG with the 6-31G basis set.

If the xc functional $E_{xc}[\rho_\alpha(X), \rho_\beta(X)]$ were exact, it would correspond to the energy of an ensemble of $S_z = +1/2$ and $S_z = -1/2$ spin states

$$\rho^{S_z=1/2}(X) = \rho_\alpha^{S_z=1/2}(X) + \rho_\beta^{S_z=1/2}(X) \tag{3}$$

$$\rho^{S_z=-1/2}(X) = \rho_\alpha^{S_z=-1/2}(X) + \rho_\beta^{S_z=-1/2}(X) \tag{4}$$

with equal weights of 1/2,

$$E_{xc}[\rho_\alpha(X), \rho_\beta(X)] = E_{xc}[\frac{1}{2}\left(\rho_\alpha^{S_z=1/2}(X) + \rho_\alpha^{S_z=-1/2}(X)\right), \frac{1}{2}\left(\rho_\beta^{S_z=1/2}(X) + \rho_\beta^{S_z=-1/2}(X)\right)] \tag{5}$$

Taking into account that the $S_z = +1/2$ and $S_z = -1/2$ spin states of the fragment X are degenerate, the ensemble energy $E_{xc}[\rho_\alpha(X), \rho_\beta(X)]$ should be equal to that of the $S_z = +1/2$

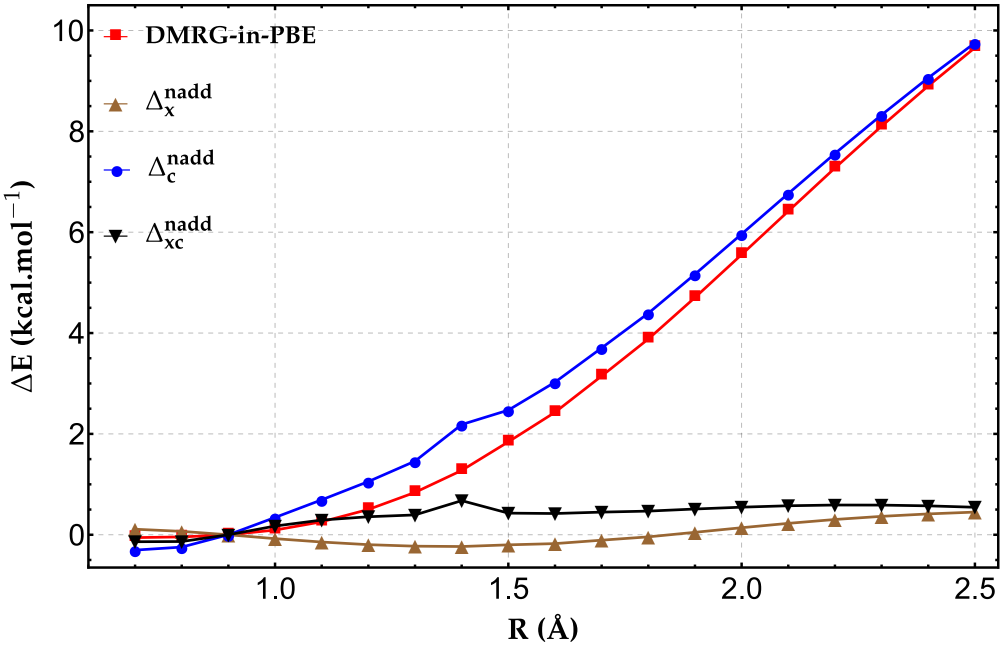

Fig. S3: Relative energy errors (in kcal/mol) of DMRG-in-PBE and DMRG-in-PBE with the nonadditive exchange (Δ_x^{nadd}), correlation (Δ_c^{nadd}), and exchange–correlation ($\Delta_{xc}^{\mathrm{nadd}}$) corrections, for 8-atom active fragment for the H_{20} hydrogen chain, benchmarked against full-system DMRG with the 6-31G basis set.

(or $-1/2$) spin state[1]

$$E_{xc}[\rho_\alpha(\mathrm{X}), \rho_\beta(\mathrm{X})] = E_{xc}[\rho_\alpha^{S_z=1/2}(\mathrm{X}), \rho_\beta^{S_z=1/2}(\mathrm{X})] \tag{6}$$

For approximate functionals, the above equality does not hold and the difference between the left and right-hand sides amounts to the fractional spin error.[1] The dissociation energy corrected for the fractional spin error, depicted in Figure S4, reads

$$\begin{aligned}\Delta_{xc}^{\mathrm{FracSpin}} &= E_{xc}^{\infty} - 2E_{xc}[\frac{1}{2}\rho_\alpha^{S_z=1/2}(\mathrm{H}), \frac{1}{2}\rho_\alpha^{S_z=1/2}(\mathrm{H})] \\ &- 2E_{xc}[\frac{1}{2}\rho^{S_z=1/2}(\mathrm{H}_9), \frac{1}{2}\rho^{S_z=1/2}(\mathrm{H}_9)] \\ &+ 2E_{xc}[\rho_\alpha^{S_z=1/2}(\mathrm{H}), 0] + 2E_{xc}[\rho_\alpha^{S_z=1/2}(\mathrm{H}_9), \rho_\beta^{S_z=1/2}(\mathrm{H}_9)]\end{aligned}$$

where it has been used that $\rho_\beta^{S_z=-1/2}(\mathrm{X}) = \rho_\alpha^{S_z=1/2}(\mathrm{X})$ and $\rho_\beta^{S_z=1/2}(\mathrm{H}) = 0$. It has been computed by running ROKS calculations for X=H and H_9 species.

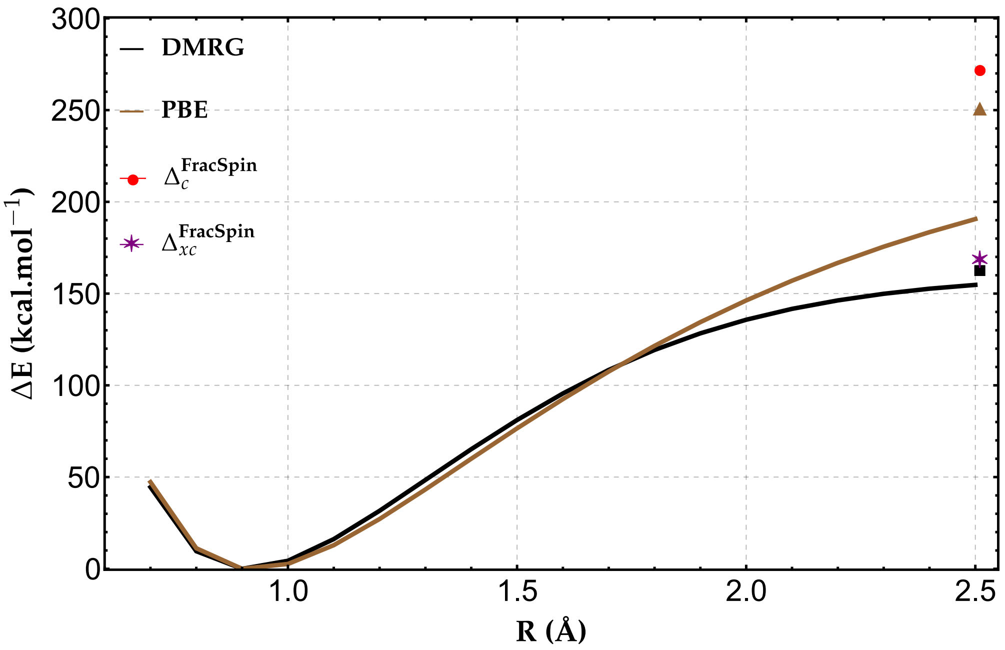

Fig. S4: Relative energy (kcal·mol^{-1}) for the H_{20} hydrogen chain, cf. Figure 1(a) in the main text, comparing RKS-PBE and DMRG. Markers denote dissociation energies for the process of fully breaking the three central H-H bonds, computed with DMRG (square), RKS-PBE (triangle), RKS-PBE dissociation energy corrected for the fractional spin error in the correlation functional ($\Delta_c^{\text{FracSpin}}$, circle), and RKS-PBE dissociation energy corrected for the fractional spin error in the exchange and correlation functionals ($\Delta_{xc}^{\text{FracSpin}}$, asterisk). Energies were obtained using the cc-pVDZ basis set.

Table S1: Absolute energies of the hydrogen chain for a given H-H bond length (in Å). All calculations were performed in the cc-pVDZ basis. Energies are listed in a.u., and DMRG calculations were performed with the DBSS procedure and TRE= 10^{-6}.

$r_{H\text{-}H}$	B3LYP	DMRG(4,10)-in-B3LYP	CAS(4,4)-in-B3LYP	DMRG
0.7	-11.32617017	-11.301103	-11.25820781	-11.25345756
0.8	-11.38169045	-11.356534	-11.31589599	-11.30910087
0.9	-11.39764576	-11.37148128	-11.33379471	-11.32438202
1.0	-11.39154785	-11.36407646	-11.32970397	-11.31740763
1.1	-11.3733303	-11.34462406	-11.31371191	-11.29856711
1.2	-11.34877215	-11.31912246	-11.29168725	-11.27396854
1.3	-11.32127998	-11.2911418	-11.26712065	-11.24720419
1.4	-11.2928809	-11.26284762	-11.24212611	-11.22047973
1.5	-11.26479733	-11.2355598	-11.21797658	-11.19515056
1.6	-11.23775795	-11.21009478	-11.19543616	-11.17209635
1.7	-11.2121916	-11.18694713	-11.17494416	-11.15163372
1.8	-11.18831162	-11.16635451	-11.15669137	-11.13430161
1.9	-11.16621283	-11.14842968	-11.14077474	-11.11985578
2.0	-11.14589699	-11.13295685	-11.12697848	-11.10804985
2.1	-11.12731172	-11.11976811	-11.11516568	-11.09859369
2.2	-11.11037503	-11.10860397	-11.10511709	-11.09125702
2.3	-11.09498597	-11.09918703	-11.09659774	-11.08550934
2.4	-11.08103945	-11.09124829	-11.08937791	-11.08108358
2.5	-11.06843045	-11.08454292	-11.08324856	-11.07777978

$r_{H\text{-}H}$	DMRG(8,20)-in-B3LYP	CAS(8,8)-in-B3LYP	DMRG
0.7	-11.28712289	-11.21182596	-11.25345756
0.8	-11.34268446	-11.26952647	-11.30910087
0.9	-11.35777494	-11.28732412	-11.32438202
1.0	-11.35059224	-11.28314385	-11.31740763
1.1	-11.33150752	-11.26716803	-11.29856711
1.2	-11.30657742	-11.24533987	-11.27396854
1.3	-11.27940772	-11.22120707	-11.24720419
1.4	-11.25220102	-11.19693078	-11.22047973
1.5	-11.2263089	-11.17382392	-11.19515056
1.6	-11.20256425	-11.15266524	-11.17209635
1.7	-11.18135047	-11.13390088	-11.15163372
1.8	-11.16308561	-11.11762839	-11.13430161
1.9	-11.14765377	-11.10387953	-11.11985578
2.0	-11.13481878	-11.09241861	-11.10804985
2.1	-11.12432108	-11.08301648	-11.09859369
2.2	-11.11584261	-11.07539547	-11.09125702
2.3	-11.10905314	-11.06926793	-11.08550934
2.4	-11.10364021	-11.0643616	-11.08108358
2.5	-11.09933576	-11.06044352	-11.07777978

Propionitrile molecule

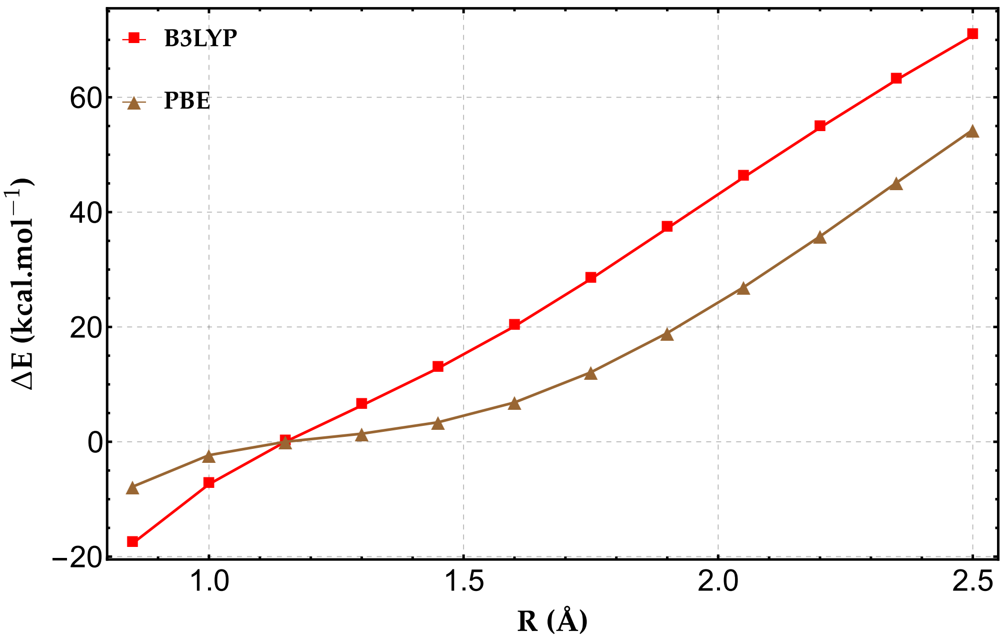

Fig. S5: Relative energy error (kcal·mol^{-1}) for the propionitrile molecule comparing B3LYP and PBE functionals. Errors are referenced to full-system DMRG energies with the cc-pVDZ basis set.

Table S2: Equilibrium geometry of CH_3CH_2CN, XYZ in Å.

C	-2.38207	-0.46087	0.01893
N	-3.18147	-0.80786	0.76930
H	-0.03176	0.93909	0.54131
C	0.02231	0.17867	-0.25262
H	0.75941	0.50795	-1.00054
H	0.38038	-0.75999	0.19640
C	-1.34723	-0.01758	-0.92251
H	-1.69162	0.92161	-1.38724
H	-1.28110	-0.76419	-1.73200

References

(1) Cohen, A. J.; Mori-Sánchez, P.; Yang, W. Fractional spins and static correlation error in density functional theory. *J. Chem. Phys.* **2008**, *129*, 121104.

Table S3: Absolute energies of CH_3CH_2CN for a given C-N bond length (in Å). All calculations were performed in the cc-pVDZ basis. Energies are listed in a.u., and DMRG calculations were performed with the DBSS procedure and TRE= 10^{-6}.

$r_{\text{C-N}}$	B3LYP	DMRG-in-B3LYP	CAS-in-B3LYP	DMRG
0.85	−171.507 897 4	−171.282 363 2	−171.111 521 2	−171.111 610 47
1.00	−171.880 723 6	−171.670 698 9	−171.494 437 6	−171.500 869 28
1.15	−171.965 237 3	−171.765 898 6	−171.587 566	−171.597 165 05
1.30	−171.933 581 4	−171.742 691 6	−171.562 922 7	−171.575 576 39
1.45	−171.861 443 1	−171.678 708 4	−171.497 740 7	−171.513 786 65
1.60	−171.781 862 7	−171.608 095 9	−171.426 259 3	−171.445 827 26
1.75	−171.708 475 6	−171.543 815 8	−171.362 579 7	−171.385 589 35
1.90	−171.646 024	−171.489 663 7	−171.311 191 5	−171.337 208 54
2.05	−171.595 446 4	−171.446 906	−171.272 426 6	−171.300 768 17
2.20	−171.556 076 1	−171.414 704 4	−171.244 897	−171.275 190 61
2.35	−171.526 392	−171.390 778 1	−171.226 471 1	−171.258 751 27
2.50	−171.504 402 5	−171.374 308	−171.214 785	−171.249 100 98
